# Long-term follow-up of thalidomide embryopathy: malformations and development of osteoarthritis in the lower extremities and evaluation of upper extremity function

**DOI:** 10.1007/s11832-014-0609-9

**Published:** 2014-10-10

**Authors:** Shadi A. Ghassemi Jahani, Barbro Danielson, Jón Karlsson, Aina J. Danielsson

**Affiliations:** 1 Department of Orthopaedics, Sahlgrenska University Hospital, Gothenburg, Sweden; 2 Department of Radiology, Sahlgrenska University Hospital, Gothenburg, Sweden; 3 Department of Orthopaedics, Kungälv Hospital, Sahlgrenska Academy, University of Gothenburg, Gothenburg, Sweden

**Keywords:** Malformation, Thalidomide, Embryopathy, Proximal femoral focal deficiency, Long term

## Abstract

**Background:**

Between 1959 and 1962, several children with multiple malformations were born after maternal intake of thalidomide during pregnancy, known as thalidomide embryopathy (TE).

**Objectives:**

The aim of this study was to evaluate the malformations, their long-term effect on the function of the extremities and the development of degenerative osteoarthritis (OA) in the lower extremities.

**Methods:**

All living persons with TE in Sweden were invited to participate in the study. Thirty-one patients were examined clinically as a part of a multi-disciplinary follow-up programme. Evaluation of upper and lower limb function was performed by validated questionnaires [Disabilities of the Arm, Shoulder and Hand (DASH) and Rheumatoid and Arthritis Outcome Score (RAOS), respectively] and radiographic appearance of lower limbs by the use of spiral computed tomography.

**Results:**

Five individuals had severe malformations of the lower limbs and proximal femoral focal deficiency (PFFD), with significantly reduced function as found on the RAOS values. Twenty-seven patients had two fully functional arms and hands, despite the fact that 8% of shoulders, 26% of elbows/forearms and 70% of hands were malformed. Loss of gripping function did not significantly affect the upper extremity function, as measured by the DASH score. Ten patients without major deformities had OA in the hips and 15 in the knees, mostly mild and with no effect on the RAOS value.

**Conclusion:**

A wide variety of malformations in the upper and lower limbs was found in the study group. Degenerative changes were found in the hips and the knees but were mostly mild and without major clinical significance. Despite upper limb anomalies that affected the fine motor skills, upper extremity function was not significantly reduced for most individuals. Individuals with PFFD along with major deformities of upper limbs had a reduced function of upper as well as lower limbs.

**Electronic supplementary material:**

The online version of this article (doi:10.1007/s11832-014-0609-9) contains supplementary material, which is available to authorized users.

## Introduction

 Thalidomide was used as a sedative drug for pregnant women in the late 1950s and at the beginning of the 1960s in many countries worldwide [1–3]. Due to its teratogenicity, thalidomide was withdrawn from the market in November 1961. The true number of affected children is unknown, but estimates are more than 10,000 affected throughout the world [1, 4]. The substance has been reported as “one of the most potent teratogenic substances in medical history” [5]. Routine screening tests of thalidomide on rodents found the substance to be non-toxic; therefore, the teratogenicity of thalidomide in humans was not anticipated [6].

Serious birth defects of the limbs were the most striking findings [5, 7–9], which included phocomelias or longitudinal deficiencies of the extremities. Many individuals had major malformations with remarkable limb shortening of the legs and arms, sometimes in combination with absent hand function. Other malformations such as those of the ears, eyes [10–12] and spine [13, 14], as well as genital and visceral defects, have also been observed in patients with thalidomide embryopathy (TE) [3, 10, 15]. Autism spectrum disorders [16] have been reported previously and craniofacial and, recently, maxillofacial malformations [17] were reported. Most individuals have malformations in multiple organ systems.

Although the teratogenicity of thalidomide in humans has been well documented [18], thalidomide was remarketed after 1965 in several countries, mostly for the treatment of multiple myeloma and erythema nodosum leprosum. As a result of this irresponsibility, and in combination with an absence of information for the mothers-to-be, children are still born today with these typical malformations [18–20]. The disaster from the early 1960s, which, shortly thereafter, was assumed to be a one-time experience, is now being repeated, leading to life-long consequences for a new generation of children.

During previous ophthalmologic studies, it had become apparent that the syndrome and its consequences were not fully mapped. Furthermore, no reports on long-term complications or outcome due to TE have previously been published. Therefore, a multi-disciplinary study was set up focusing on the individuals with TE born in Sweden in the late 1950s and early 1960s. The aim of this study was to evaluate only the orthopaedic parts of the multi-disciplinary project, i.e. the function and radiological appearance of the limbs in individuals with TE. We aim to answer the following questions: What types of malformations occurred in the lower extremities? Did a grasping function of the upper extremity exist? Did the malformations of the upper or lower extremities affect the function as measured by validated specific questionnaires? Have osteoarthritic changes in the hips and knees developed, and, if so, did they have any clinical impact?

## Materials and methods

The Swedish Association of Individuals with Thalidomide Embryopathy includes all individuals diagnosed with TE. This association was established during the 1960s when legal processes were undertaken. Approximately 150 children with TE were born in Sweden between 1959 and 1962. One-third died during childhood, most of them in the neonatal period due to severe malformations [10]. Further individuals were later diagnosed with TE and some individuals later died of different causes. Due to too much previous undesirable contact to the members of the association, strict legal rules were formed as a protective measure.

All 108 members of the association at the time of the study were invited to participate in the present study. Twenty-four patients, who previously through the association had rejected contact for studies of any kind, had to be excluded. Thus, 84 patients received an invitation to participate in the investigation. Thirty-three persons did not reply to the invitation, but due to the existing rules of the association, no reminders of the invitation letter were allowed to be sent out. Eighteen patients rejected participation and 33 patients agreed to participate. Two of the patients who accepted the invitation were unable to participate due to severe neurological disability; one was institutionalised, while the other suffered a stroke just before the examination was due to take place. Finally, 31 participants (13 female and 18 males) with a mean age of 45.8 [standard deviation (SD) 1.1] years were examined.

The study was multi-disciplinary and represents, besides orthopaedics, also ophthalmology, otolaryngology, speech pathology, dentistry, neuropsychiatry and radiology, which have been [11, 17, 21] or will be presented separately. Patients were examined at the Sahlgrenska University Hospital in Gothenburg and all the examinations were performed on one day. All patients were living in Sweden at the time of the investigation, apart from one who came from the USA and two who came from Norway.

### Clinical examination

All participants underwent a full clinical evaluation by the same orthopaedic surgeon SAGJ, including joint and spine mobility, as well as a full neurological examination. The existence and location of malformations, deformities and joint contractures were noted. The Lachman test was used for the evaluation of sagittal knee laxity. Information relating to previous medical history was collected from the patients.

Evaluation of the upper extremities focused on hand and grip function, rather than a description of each malformation; a hand was defined as the existence of a palm (complete or part of) with more than two fingers. An anatomical pincer grasp was defined as the existence of a functional thumb with opposition, i.e. not necessarily an original thumb. Grip function was defined as any hand with the ability to hold items, regardless of an existing pincer grasp and regardless of where the hand was located on the upper extremity, as long as it served as a gripping tool, which also included a release mechanism. In order to perform a general evaluation of the physical abilities, individuals were grouped in terms of the severity of the total musculoskeletal burden. A major malformation of an extremity was considered when considerable shortening or deformity existed, but limbs with malformation(s) of the fingers or toes only, were not considered as such. Individuals with up to three extremities with major involvement were regarded as one group and those with all four extremities affected by major malformations were grouped together.

### Radiography and spiral computed tomography

Due to the focus of the study on the occurrence of osteoarthritis (OA) of the lower extremities, the lower limbs, including the feet, were examined with computed tomography (CT). In order to reduce the radiation dose, a spiral CT was performed. Due to the radiation dosage, the spine and upper extremities were not examined. The examinations could only be performed in the supine position; therefore, no load-bearing images could be obtained. Care was taken that the patellae were facing upwards and centrally. The sliding, two-dimensional simultaneous radiographic technique (the EOS system) was not available on the market at the time of planning of this study.

The CT examination was performed with 16-section equipment (LightSpeed Pro, GE Healthcare, Milwaukee, WI, USA). Following coronal and sagittal tomogram acquisition, an axial helical scan was obtained without contrast injection. The following parameters were used: 140 kV, 95–198 mA, 893 ms, single collimation width 0.625 mm, total collimation width 10 mm, minimum slice thickness 1.25 mm, spiral pitch factor 0.9375 mm, collection field of view (FOV) 500 mm, reconstruction FOV 400–462 mm and bone kernel. The total radiation dose given to the patient was registered and none exceeded 2.198 mGy/cm.

Reconstructed images in the axial and sagittal planes were obtained at the scanner. The slice thickness in the axial, coronal and sagittal planes was 2.5, 3 and 3 mm, respectively. In all subjects, axial thin-slice source images were recalled from the picture archiving and communication system (PACS) and loaded onto a commercial workstation (General Electric Medical Systems, Centricity Radiology, RA 600v8.0). In some patients, new reconstructions were created at another work station (General Electric Advantage Workstation 4.2.4, LightSpeed Pro, GE Healthcare, Milwaukee, WI, USA) using the thin axial slices.

**Fig. 1:**
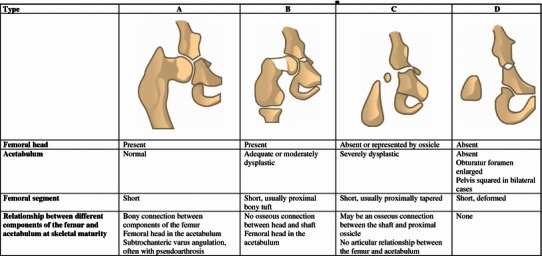
Classification of proximal femoral focal deficiency (PFFD) according to Aitken [24]

Reconstructed images were first evaluated in consensus by two experienced radiologists (JF and BD) and, thereafter, evaluated together by two orthopaedic surgeons (SAGJ and AD). The presence of any skeletal malformation or OA in the hip, knee, ankle, mid-foot, metatarsophalangeal and toe joints was assessed and recorded. For the classification of proximal femoral focal deficiency (PFFD), the Gillespie classification [22] was used; group I includes those with a congenital hypoplastic femur in which the hip and knee could be made functional and group II includes those with true PFFD, where the hip joint was never normal. The Aitken classification [23–25] was used for a more thorough description of the status of the femoral head and neck (Fig. 1).

The classification of OA was based on the classification system developed by Ahlbäck [26], graded from I to V. As the CT examination was performed without weight-bearing, an adjusted scale was used for all the joints in the lower limbs; no sign of OA was recorded as grade 0, the existence of only a few osteophytes or/and reduced cartilage height as grade 1 (‘mild’) and the existence of osteophytes and cysts as grade 2 (‘severe’). The whole hip joint was evaluated for OA, while the knee was evaluated in three different zones (lateral, medial and patello-femoral joint). The degree of OA could not be evaluated in case of malformation or absence of the joint.

**Fig. 2:**
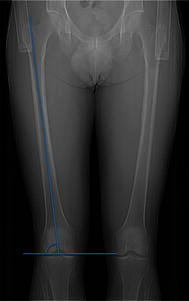
Measuring of varus or valgus angles of the distal femur using the tomograms. Varus or valgus angles of the distal femur were measured between the long axis of the femur and the joint surface of the distal femur. As the spiral computed tomography (CT) was obtained only in the lying position, a proper evaluation of mechanical and anatomical axes, which should be performed in an erect position for ambulating individuals, could not be performed

Using the tomograms, varus or valgus angles of the joint surfaces of the distal femur were measured between the long axis of the femur and the joint surface of the distal femur (Fig. 2). As the spiral CT was obtained only in the lying position, a proper evaluation of mechanical and anatomical axes, which should be performed in an erect position for ambulating individuals, could not be performed.

### Functional assessment

The evaluation of lower extremity function was performed according to the Rheumatoid and Arthritis Outcome Score (RAOS), intended to evaluate symptoms and functional limitations in persons with problems of the lower extremities [27]. It is a self-administered 42-item instrument assessing five separate patient-relevant dimensions; pain, other symptoms like stiffness, swelling and range of motion, activities of daily living (ADL), sport and recreational (Sport/Rec) and lower limb-related quality (QoL). Each item is answered as no, mild, moderate, severe or extreme (scored as 0–4, respectively). Each of the five subscale scores is calculated as the sum of the items included and raw scores are transformed to a 0 to 100, worst to best, scale.

The Disabilities of the Arm, Shoulder and Hand (DASH) Outcome Measure was used for the assessment of upper extremity function [28]. This questionnaire evaluates disease-specific quality of life in terms of physical function/symptoms in upper limb disorders. It includes the disability/symptom scale (the DASH score, all 30 items) and the optional high-performance sport/music and work scales (four items each). Scoring ranges from 1 (no difficulty to perform an activity) to 5 (impossible to perform), and at least 27 out of 30 questions need to be answered. The scores are calculated as [(sum of *n* answers/*n*) − 1] × 25 and the result varies from 0 (best performance) to 100 (worst performance). The results were compared to the American norm scales for individuals aged 30–49 years [29], as no Swedish norms have yet been published.

### Ethics

The Human Research Ethics Committee at the Sahlgrenska Academy at Gothenburg University approved the study.

### Statistical analysis

Distributions of variables are given as means, SDs and ranges. For comparisons between two groups, the Mann–Whitney non-parametric *U*-test was used for continuous variables. When comparing two groups, the Chi-square test for ordered categorical variables was used.

All the significance tests were two-tailed and conducted at the 5 % significance level.

## Results

Eighteen (58 %) men and 13 (42 %) women with a mean age of 45.8 (SD 1.1) years were examined. Fifteen patients reported having a variety of other diseases, of which asthma (3) and hypertension, sleep apnea syndrome and migraine (each 2) were the most commonly occurring. No patient was found to have a major deformity of the spine at the clinical examination.

### Findings in the upper extremities

Reduced abduction, less than 90°, was found in 11 shoulders. Another five shoulders had malformations.

An extension deficit of more than 10° was found in 15 elbows and flexion of less than 90° in ten elbows. Three of these patients had a reduction in both flexion and extension and one of them was bilaterally affected.

Eleven patients displayed radial bowing of the forearm, indicating radial aplasia or hypoplasia.

The most frequent deformities of the upper extremities were found in the hands, with 43 (69.4 %) of the hands being affected.

A bilateral pincer grasp was found in 11 patients, indicating no major malformations of hand anatomy responsible for this function. Twenty patients had an anatomical pincer grasp in one or no hand.

Twenty-seven patients had two arms with sufficient grip and release function, even though not all of them had an existing pincer grasp due to malformation of the hand or lower arm. The remaining four individuals were severely affected; two patients had only one right-sided fully functional arm and a short left arm without grip function, and the other two patients with bilateral short upper limbs did not have any grip function on any side. One of these limbs was a single finger attached directly to a left shoulder.

Seventeen surgical procedures of the upper extremity had been performed, 15 of those on the hand or wrist and 11 before the age of 18 years.

Only one woman with reduced grip function used her feet for help in daily activities occasionally. Two individuals used hand orthoses occasionally.

### Findings in the lower extremities

#### Patients with major anomalies

**Fig. 3:**
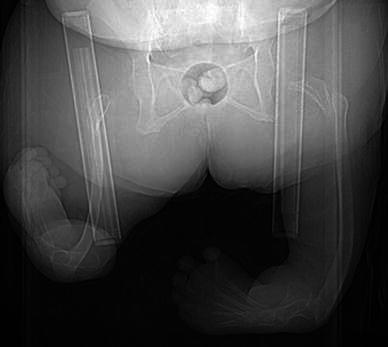
Patient with proximal femoral focal deficiency (PFFD), anteroposterior (AP) view. Bilateral severe dysplasia of the acetabulum and short femur with no apparent hip joint and tibial aplasia, right foot in calcaneovalgus position, while the left has a clubfoot deformity. The longitudinal bars are used to position the patient during examination

Five (16.1 %) of the 31 patients were diagnosed with PFFD (Table 1, Fig. 3). Five of these nine lower limbs also had fibula/tibia hypo- or aplasia; only one of the lower extremities seemed to be of somewhat normal length. One individual was amputated below the knee, did not use any prosthesis and used an electric wheelchair. Another two individuals used a whole-leg prosthesis due to limb shortening. None had undergone a Van Nes rotationplasty. A wheelchair was used most of the time by three individuals (patients II, III and V), while two managed walking with or without crutches (patients I and IV) (Table 1).

**Table 1:** Description of the five individuals with thalidomide embryopathy (TE) who had proximal femoral focal deficiency (PFFD) as found by clinical examination and radiological evaluation of the lower extremities

Patient no.	I		II		III		IV		V	
	R	L	R	L	R	L	R	L	R	L
Lower extremities										
Hip/femur										
PFFD, type according to Gillespie/Aitken^a^	II/B		II/D	II/D	II/C		I/C	II/C	I/D	II/D
Dysplastic, dislocated hip joint		X								
Deformed femoral head						X				
Extension deficit >10 °	X						X		X	X
Knee										
Hypoplastic lateral femoral condyle^b^		X							X	X
Intercondylar notch hypoplasia		X				X	X			
Dislocated patella							X			
Severe osteoarthrosis of the knee							X			
Remarks	No deformity		No deformity	No deformity		Exarticulated	Varus			
Lower leg										
Fibula aplasia										
Tibia hypoplasia/aplasia			X	X	X			X		X
Foot										
Equinus and varus position				X			X	X	X	X
Equinus and cavus position	X	X								
Calcaneovalgus foot			X							
Calcaneus only existing bone					X					
Remarks						No foot				
Significant limb shortening	X		X	X	X	X	X	X	X	X
Upper extremities										
Hand										
Hypoplastic thumb	X				X	X				
Absence of the thumb		X	X	X						X
Missing/extra finger/s			X	X					X	
Other										
Radial bowing of the forearm					X	X				

*R* right, *L* left

^a^According to Gillespie and Aitken’s classification, Fig. 3

^b^The proximal tibia was adopted to the hypoplastic lateral condyle in all patients with this finding

Malformations of the feet were found in five of the lower limbs in these five patients. All five patients with PFFD had several malformations of the upper extremities.

#### Patients without major anomalies

Twenty-six patients (83.8 %) did not have any major congenital deformities of the lower limbs (Table 2).

**Table 2:** Description of the 26 individuals with thalidomide embryopathy (TE) that did not show any signs of proximal femoral focal deficiency (PFFD) as found by clinical examination and radiological evaluation of the lower extremities

	*n* (%) or mean (SD)/(range)
Hip/femur	
Malformations	
Deformed femoral head^a^	34 (65.4)
Previously performed surgery (no. of procedures)^b^	
Acetabuloplasty	1
Hip arthroplasty	1
Unspecified surgery on the hip	1
Knee	
Malformations	
Hypoplastic lateral femoral condyle	27 (51.9)
Proximal tibia adapted to the hypoplastic lateral femoral condyle	20 (38.5)
Intercondylar notch hypoplasia	26 (50)
Valgus deformity (°) of the knee in patients with:^c^	
Normal lateral femoral condyle	7.9 (3.0)/(1.8–12.5)
Hypoplastic lateral femoral condyle	10.6 (2.8)/(6.3–17.1)
Lachman test positive at clinical examination	5 (9.6 %)
Previously performed surgery	
Cruciate ligament reconstruction	1
Foot/ankle	
Malformation	0
Previously performed surgery	
Tendon harvesting of the foot	1

^a^See Fig. 4

^b^Performed in the same patient

^c^*p* = 0.08 for comparison with normal or hypoplastic lateral femur

**Fig. 4:**
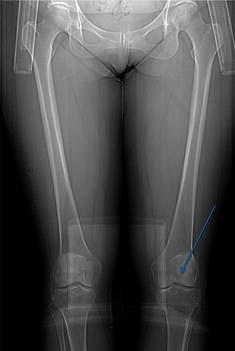
Patient with slightly deformed hips and knees with lateral femoral condyle hypoplasia and hypoplastic intercondylar notch (see *arrow*)

A slightly deformed femoral head was found in 34 hips (65.4 %, Fig. 4).

Knee abnormalities with a hypoplastic lateral femoral condyle were found in 27 (51.9 %) knees; in 20 of these, an adaptation of the proximal tibia was found (38.5 % of the knees). Patients with hypoplastic lateral femoral condyles had a significantly larger valgus angle of the knee compared with those without, 10.6° vs. 7.9° (*p* = 0.008). Twenty-six of these patients (50 % of all knees) also displayed a hypoplastic intercondylar notch (Fig. 4). Five patients had knee instability at clinical examination, indicating insufficient cruciate ligament function, but only two of these patients also had a hypoplastic intercondylar notch radiographically.

All were walking independently without any support.

### OA of the joints in the lower extremities

#### OA in the five patients with PFFD

Most of the patients with PFFD had severe joint malformations in the lower extremities, which made the evaluation of OA impossible. Two patients with an anatomically recognisable hip and knee, respectively, showed signs of severe OA (Table 3).

**Table 3:** Occurrence and location of osteoarthritis (OA) in the lower extremities in the 26 individuals with thalidomide embryopathy (TE) but without proximal femoral focal deficiency (PFFD)

	Grade 0: no OA	Grade 1: mild OA	Grade 2: severe OA
Hip joint			
Right ^a^	15 (57.7)	5 (19.2)	5 (19.2)
Left	18 (69.2)	4 (15.4)	4 (15.4)
Occurrence of OA on any side	16 (61.5)	10 (38.5)	
Bilateral OA of all grades		7 (26.9)	
Knee joint			
Right			
Medial	14 (53.8)	5 (19.2)	7 (26.9)
Lateral	22 (84.6)	4 (15.4)	–
Patello-femoral	20 (76.9)	5 (19.2)	1 (3.8)
Left			
Medial	15 (57.7)	7 (26.9)	4 (15.4)
Lateral	25 (96.2)	1 (3.8)	–
Patello-femoral	25 (96.2)	1 (3.8)	–
Occurrence of OA on any side	11 (42.3)	15 (57.7)	
Bilateral OA of all grades		9 (34.6)	

Values are expressed as *n* (%)

One patient previously had a calcaneus fracture with dislocation and showed OA of the subtalar joint. The fracture was considered to be the major factor for OA in this patient, who was, therefore, excluded from the analysis

^a^Evaluation in one person not possible due to hip replacement

Four patients had osteoarthritic changes in the first metatarsophalangeal joint, and two of them had bilateral changes, one severe and one mild OA. One patient had bilateral mild OA of the II–V metatarsophalangeal joints.

#### OA in the 26 patients without PFFD

The occurrence of OA in the hip and knee joints of the 26 patients without PFFD is presented in Table 3. Bilateral hip OA was found in seven (26.9 %) and OA of the knee in nine (34.6 %) patients. Only three patients displayed severe osteoarthritic changes in the first metatarsal joint. None had any OA in the ankle and only two mid-foot joints showed severe OA, one of those possibly due to a previous calcaneus fracture. The osteoarthritic changes that were found were mostly regarded as mild.

### Function

#### RAOS

The results of the RAOS scoring for all 31 individuals are detailed in Table 4. The individuals with PFFD had significantly lower score in terms of ADL (56.0 vs. 87.3, *p* = 0.007), sport and recreation (9.0 vs. 72.1, *p* = 0.001) and quality of life (40.2 vs. 71.2, *p* = 0.015) compared with those without this malformation.

**Table 4:** Results of the Rheumatoid and Arthritis Outcome Score (RAOS) values representing the subjectively evaluated function of the lower extremities in 31 individuals with thalidomide embryopathy (TE)

	*N*	All	Occurrence of PFFD		
			No (*n* = 26)	Yes (*n* = 5)	*p*-Value
Pain	31	78.5 (20.6)/(36–100)	80.4 (19.8)/(36–100)	68.1 (23.6)/(47–100)	0.31 (n.s.)
Symptoms	31	78.6 (17.9)/(32–100)	80.2 (18.0)/(32–100)	70.0 (16.5)/(50–89)	0.20 (n.s.)
ADL	30^a^	83.1 (19.7)/(40–100)	87.3 (17.2)/(41–100)	55.9 (12.7)^a^/(40–71)	0.0076
Sport and recreation	31	61.9 (36.4)/(0–100)	72.1 (30.1)/(10–100)	9.0 (9.6)/(0–25)	0.0007
Quality of life	31	66.1 (26.2)/(19–100)	71.2 (24.8)/(19–100)	40.0 (16.9)/(25–69)	0.016

Values are expressed as mean (SD)/(range)

*PFFD* proximal femoral focal deficiency, *ADL* activities of daily living

^a^One patient did not answer all items

A reduced score for ADL was found for those with OA of either hip (79.0) compared to those without OA (93.5, *p* = 0.036). Apart from this subscale, OA of the hip or knee did not correlate with the RAOS values, i.e. the function of the lower limbs.

#### DASH

The upper extremity function, as measured by the DASH score, is presented in Table 5. A loss of a pincer grasp or a general functional grip (in one or both hands) was not found to significantly affect the upper extremity function, as measured by the DASH score. However, individuals with all four limbs having major malformations showed a significant reduction of the ground score of DASH compared with those with fewer limbs affected (25.5 vs. 14.3, *p* = 0.015).

**Table 5:** Results from the Disabilities of the Arm, Shoulder and Hand (DASH) questionnaire and subscores presenting the subjectively evaluated function of the upper extremities in 31 individuals with thalidomide embryopathy (TE)

	*N*	All	Anatomical pincer grasp			Functional grip function			No of extremities with major malformations		
			In one or no hand (*n* = 20)	In both hands (*n* = 11)	*p*-Value	In one or no hand (*n* = 4)	In both hands (*n* = 27)	*p*-Value	0–3 (*n* = 16)	4 (*n* = 15)	*p*-Value
Total DASH score	31	20.5 (15.6)/(0–73)	24.2 (15.4)/(0–73)	13.9 (14.5)/(0–46)	0.057 (n.s.)	31.9 (30.8)/(3–73)	18.8 (12.2)/(0–46)	0.58 (n.s.)	14.3 (12.1)/(0–46)	25.5 (17.3)/(0–73)	0.015
Work score	25^a^	13.3 (17.8)/(0–69)	15.4 (18.9)/(0–69), *n* = 18	8.14 (14.9)/(0–38), *n* = 7	0.26 (n.s.)	4.2 (7.2)/(0–13), *n* = 3	14.5 (18.5)/(0–69), *n* = 22	0.31 (n.s.)	10.1 (13.1)/(0–38), *n* = 12	15.4 (21.4)/(0–69), *n* = 13	0.62 (n.s.)
Sports/music score	13^a^	22.1 (24.2)/(0–69)	22.0 (22.2)/(0–56), *n* = 6	22.4 (27.5)/(0–69), *n* = 7	0.88 (n.s.)	18.8 (26.5)/(0–38), *n* = 2	22.7 (25.0)/(0–69), *n* = 11	0.834 (n.s.)	19.5 (26.6)/(0–69), *n* = 8	21.9 (22.3)/(0–56), *n* = 5	0.45 (n.s.)

Values are expressed as mean (SD)/(range)

Major malformation of an extremity was considered when considerable shortening or deformity existed. Existing malformation(s) of the fingers or toes ONLY were not considered a major malformation

^a^*n* = all individuals did not answer all questions

Results for the full group of individuals with TE were compared with the age-related norm scales. The total score was significantly lower for the TE group compared to the norm scales, mean/SD 20.5/15.5 vs. 14.0/15.4, (*p* = 0.035), but no differences were found for the subscores work or sports/music.

## Discussion

Individuals with TE often have multiple anomalies, including musculoskeletal, which was the major reason for starting the present study as a multi-disciplinary follow-up with adult individuals. Results regarding dental status, oral function and facial palsy have been published [11, 17, 21]. In this orthopaedic part of the study of adult individuals with TE, malformations in both upper and lower extremities were found in all 31 participants. A limitation of this study is that less than 40 % of the study group accepted the invitation to participate.

The tragic circumstances surrounding the thalidomide disaster and the apparent anomalies with sometimes lack of all four extremities did result in considerable attention in the media for persons with TE. The lengthy legal processes in combination with a heavy burden of the parents in combination with more or less problematic activities of daily life has made many of these individuals renounced participation in a study or project. This approach has been mainly adopted by those individuals who have the most serious injuries, where attention previously had been great and where long journeys of participation become too difficult to implement. The legal construct of the association for individuals with TE made it impossible to remind or re-invite non-answering invitations, which also contributed to the low number of participants. With this background, this is the best follow-up rate that could be achieved. Given that children continue to be born today with these injuries, we consider it of great value to present the results, despite the limited follow-up rate.

CT was performed on the lower extremities, which made description of the anomalies possible. Our original plan to evaluate degenerative changes over time by comparing with older radiographs failed to implement, since not all individuals had been radiographically examined previously and many images that really existed could not be retrieved.

The group of five individuals with PFFD all had severe malformations of the lower legs (such as tibia or fibula hypo- or aplasia), as well as in the upper extremities. Most had difficulties in walking and/or needed orthoses/prostheses. The remaining individuals did not have any severe malformations of the lower extremities, but, among these, half of the lower limbs showed radiological signs of a notch deformity, i.e. hypoplasia of the intercondylar part of the distal femur. This has been reported to appear as a sign of congenital insufficiency or absence of cruciate ligaments [30, 31], and has also been previously reported in children with congenital hypoplasia of the femur [32] and of the lower extremity [33]. However, only two participants also showed sagittal knee instability at clinical examination, indicating insufficient cruciate ligament function, and another patient had previously undergone anterior ligament reconstruction surgery. Half of the lower limbs showed a hypoplastic lateral femoral condyle with a significantly increased valgus angle of the knee. The clinical importance of these findings is, however, unclear.

OA of the hip was found in nearly 40 % and OA of the knee in 60 % of the individuals. Only individuals without major deformities were included in this analysis, as major deformities might affect the development of OA. Comparisons of the OA rates in the hip and knee towards the healthy population are problematic, as the previously reported prevalence of OA in these joints show large variations. Furthermore, the findings are based on either symptoms or radiographic examinations, or a combination of both. According to the national BOA report (Better management of patients with OsteoArthritis) [34], 15 % of individuals in Sweden between the ages of 35 and 55 years have pain in one knee, considered to be most probably due to OA in the affected knee. The same relationship exists for hip OA [34]. In studies based on symptoms in combination with a radiological diagnosis, the prevalence of hip OA in the whole population varies in the range 1.9–8.3 % for men and 2.5–11.1 % for women and, for OA of the knee, between 4.7–15.8 and 6.6–31.8 %, respectively [35, 36]. In the recent study by Guillemin et al. in 2011 [35], age-based prevalence was reported. The age group 40–49 years, equivalent to our study group, had a prevalence of OA of the hip of 0.96 % for men and 0.76 % for women, while the prevalence of OA of the knee was 2.09 % and 1.64 %, respectively. A previous study concerning the epidemiology of OA in Australia [37] reported a somewhat higher prevalence of OA in general of the knee or hip; 5 % below the age of 40 years and 10 % between the ages 45 and 65 years. The OA frequency of both the hip, 38 %, and of the knee, 58 %, of the present study are clearly greater than the previously published results from the general populations, and this is despite the fact that CT, often regarded as inferior to magnetic resonance imaging (MRI) for the diagnosis of OA, has been used.

The study did not aim to find the reasons for the development of OA, merely to reflect the clinical impact. It appears that the clinical impact of OA was low, as none of the values of the RAOS were significantly reduced. One reason for this might be that approximately half of the OA findings were mild. Only ADL were affected significantly by the occurrence of hip OA, and then with a decrease of the mean of approximately 15 points. It is, however, difficult to assess the relevance of this, as norm scales and an evaluation regarding the level of clinically meaningful difference has not yet been published.

The individuals with PFFD who also had severe malformations of the lower leg had a significantly reduced function as measured by the RAOS. Prostheses and/or wheelchairs were used by most patients and ADL, sports and recreation, as well as quality of life, were significantly reduced. Bremander et al. [27] reported on RAOS scoring in 1,525 patients with rheumatoid arthritis (RA) with a mean disease duration of 18 years. The ADL and sport and recreation scores were lower (11 and 21 points, respectively) in the arthritis group than in the present study group, but the RA patients were approximately 10 years older however.

The majority of the participants had some kind of functional arm/arms, i.e. some type of grip function, although approximately only half of the patients had a bilateral proper pincer grasp and some had very short upper limbs.

For evaluation of upper extremity function, the DASH score was used. Even though this questionnaire has been used in Sweden, no nation-based norm scales have been published. We, therefore, used the American norms instead [29]. The ground score was significantly reduced in the TE group compared with these norms, with a score difference of 8. However, Gummesson et al. in 2003 [38] stated that a 10-point difference in the mean DASH score could be considered to reflect a minimally important change. This means that the difference between the whole TE group and the norms did not reach a level that would reflect a clinically meaningful difference.

Furthermore, no significant differences were found in terms of the DASH score between subgroups with loss of either the pincer grasp or the functional grip function. These findings support that, even though many patients had substantial congenital defects (as evaluated by the ‘anatomical pincer grasp’), most individuals could use the rest of the arm in some other way and still got a satisfactory grip function (as evaluated by the ‘functional grip function’), thereby obtaining an acceptable performance level. None of the individuals in this study group needed their lower limbs as an alternative gripping ability.

For individuals with major deformities in all four extremities, a reduction of upper extremity function was noted however. The reason for these findings is not clear; one might be that having normal lower extremities makes positioning of the body easier, which results in better reaching ability; another might be that the DASH questionnaire is too rough to detect differences regarding fine motor skills.

This is, according to our knowledge, the only long-term follow-up in adulthood of individuals with TE. Despite the fact that this group does not include a majority of the original group and that the group might be skewed regarding the severity of the deformity, the 31 individuals who took part in the study do reflect the full spectrum of malformations, which makes analysis of existing malformations and a subsequent evaluation of the function possible. We, therefore, found it worthwhile to present these results. When searching the literature for other comparative studies, using the search words “congenital malformation”, “congenital limb deficiency”, “outcome”, “long term” and combinations thereof, no study on complex congenital syndromes could be found. One long-term study was published in 2013 concerning Van Nes rotationplasty in patients with congenital PFFD [39]. This study includes individuals with PFFD only, in contrast to the present study group with complex and mixed anomalies, and the validated outcomes measures used in that study were not similar to those in the present study. This makes comparisons of function between groups problematic.

In conclusion, in this group of individuals with TE, a wide variety of congenital malformations in upper as well as lower limbs was found. Degenerative changes of the hips and the knees occurred more frequently than normal, but were mostly mild and had little clinical significance. The function in upper limbs was not significantly reduced for most individuals, despite the affection of the fine motor skills due to the anomalies in the upper extremities. Individuals with PFFD who had major deformities in the upper limbs as well had reduced function in upper as well as lower extremities.

## Electronic supplementary material

Below are the links to the electronic supplementary material.

Supplementary material 1 (PDF 265 kb)

Supplementary material 2 (PDF 63 kb)
