# Supplementary figures and images for: Long-term follow-up of thalidomide embryopathy: malformations and development of osteoarthritis in the lower extremities and evaluation of upper extremity function
Source: J Child Orthop. 2014 Oct 10;8(5):423–33. doi: 10.1007/s11832-014-0609-9 (PMC4391049; doi:10.1007/s11832-014-0609-9)

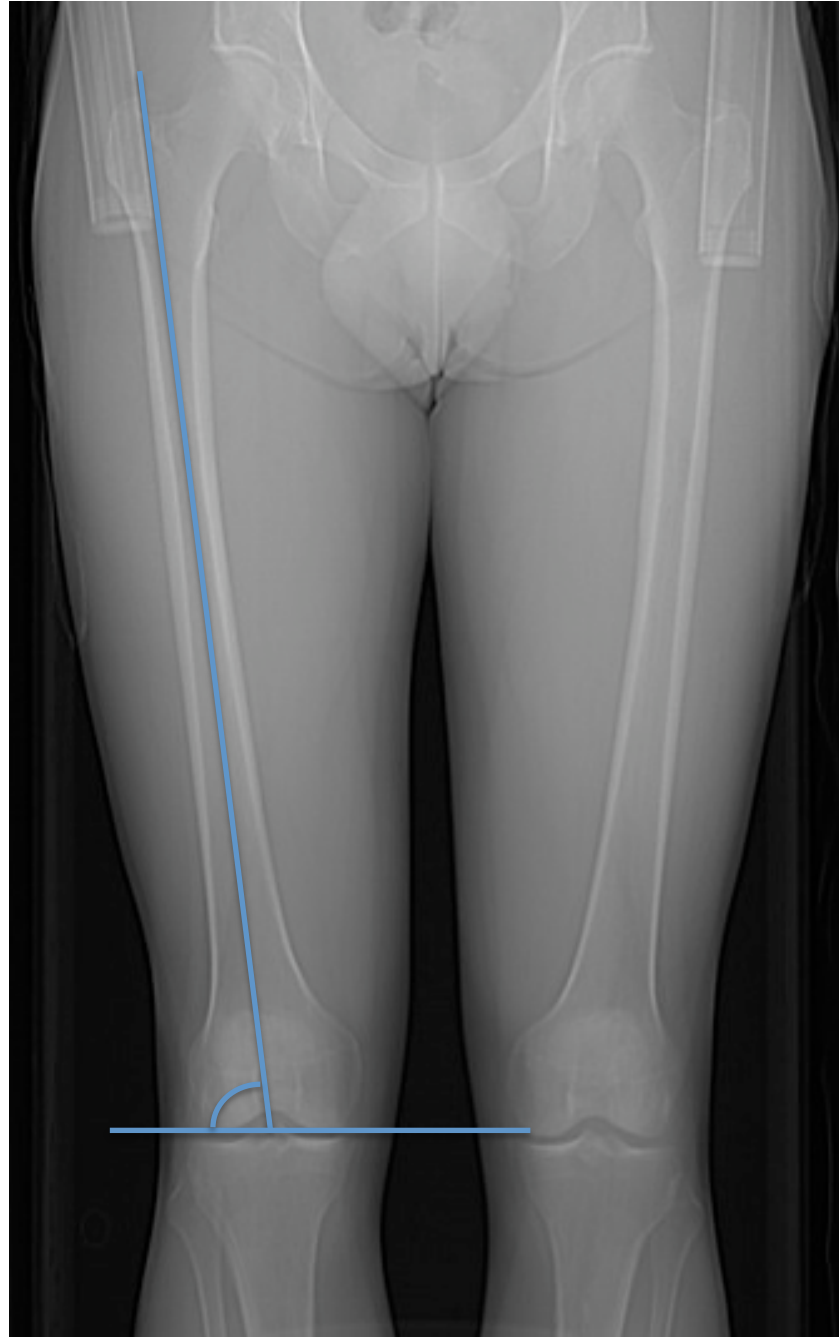

Supplement: Supplementary file 1 — Supplementary material 1 (PDF 265 kb) [file 11832_2014_609_MOESM1_ESM.pdf]

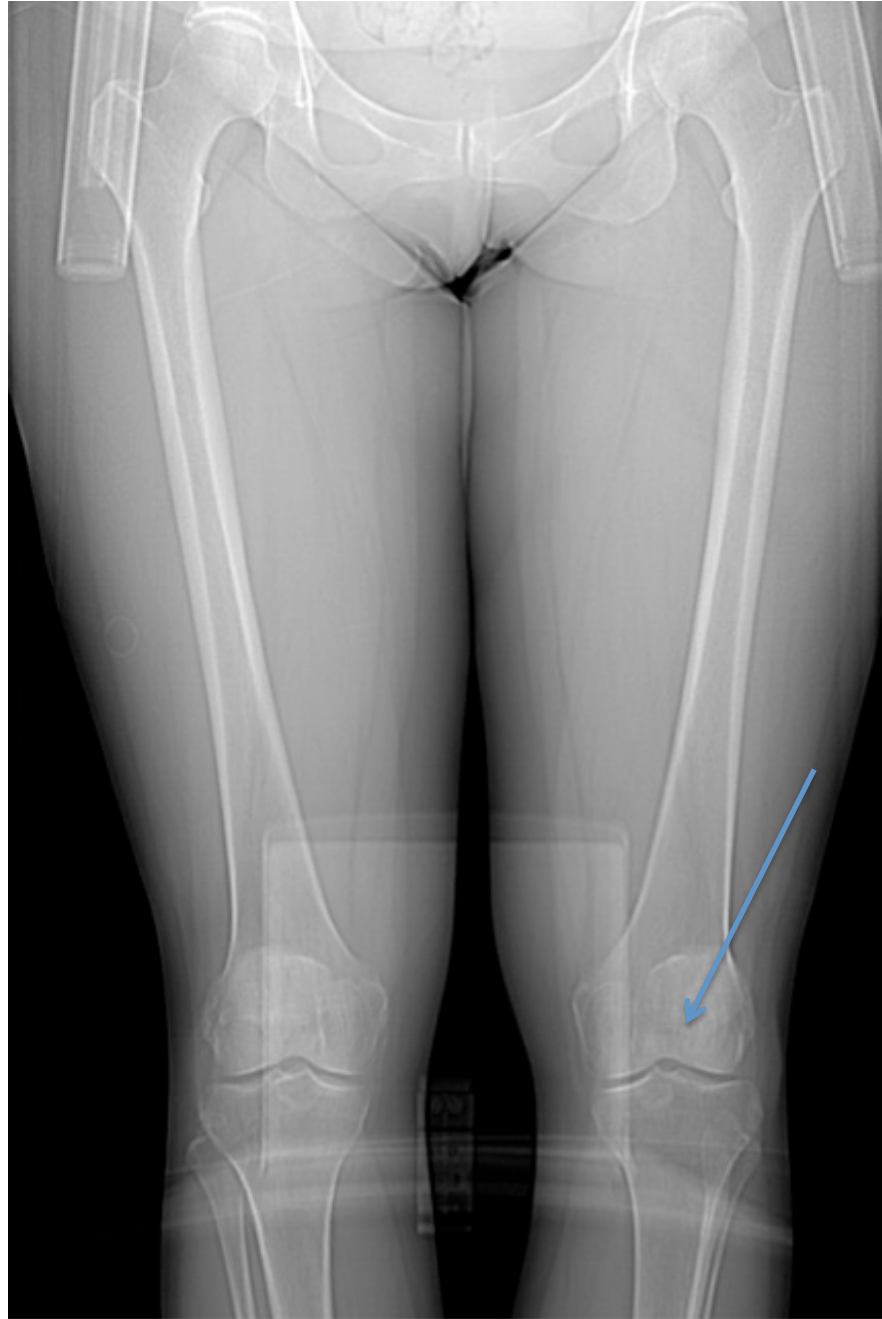

Supplement: Supplementary file 2 — Supplementary material 2 (PDF 63 kb) [file 11832_2014_609_MOESM2_ESM.pdf]
